# Reflections on qualitative data analysis training for PPI partners and its implementation into practice

**DOI:** 10.1186/s40900-019-0156-0

**Published:** 2019-08-14

**Authors:** Alison Cowley, Margaret Kerr, Janet Darby, Pip Logan

**Affiliations:** 1 Institute of Nursing & Midwifery Care Excellence, Nottingham University Hospitals NHS Trust, Hucknall Road, Nottingham, NG5 1PB UK; 2 Division of Rehabilitation, Ageing and Well-being, School of Medicine, University of Nottingham, Queen’s Medical Centre, Nottingham, NG7 2UH UK; 3 Patient and Public Involvement Partner C/O School of Health Sciences, University of Nottingham, Queen’s Medical Centre, Nottingham, NG7 2UH UK; 4 Nottingham CityCare Partnership, 1 Standard Court, Park Row, Nottingham, NG1 6GN UK

**Keywords:** Co-production, PPI, Qualitative research, Training

## Abstract

**Plain English summary:**

Service users should be involved in every part of the research process, including analysis of qualitative research data such as interviews and focus groups. To enhance their participation, confidence and contributions, training and support for both the ‘professional’ researcher and lay member of public is essential. Historically this has taken a number of forms from short 1 day training sessions through to training spread out over several months. There currently is limited guidance on the quantity and content of such training sessions for Patient and Public Involvement (PPI) Partners. This paper discusses and explores the content and delivery of qualitative analysis training held over two sessions of 3 h duration to members of a University PPI group. The training was designed by experienced qualitative researchers and PPI partners based on available literature and research expertise. Training included the theory of qualitative research methods, and practical qualitative analysis coding skills. These skills were developed through the use of ‘mock’ interviews which participants practiced coding in supportive group sessions. Their feedback on the training is provided. One of the PPI partners subsequently went onto code data with a researcher working on a funded research study, and has reflected on both the training sessions and the subsequent analysis of the data. These reflections have been supplemented by reflections of the researcher who worked alongside the PPI partner, revealing that the process challenged perspectives and helped them view data through a service users eyes. A positive working relationship was central to this.

**Abstract:**

**Background:**

Service users should be involved in every part of the research process to ensure that interventions are fit for those whom they are intended to help. Involving service users in analysing qualitative data such as focus groups and interviews has been recognised as particularly valuable. Older people have frequently been less involved in these initiatives. A wide range of training programmes have been proposed but there is currently limited guidance on the quantity and content of training sessions to support training Patient and Public Involvement (PPI) Partners. This paper discuses and explores the content and delivery of qualitative data analysis training to members of a University PPI Group.

**Body:**

Existing literature on PPI in qualitative data analysis was reviewed by the research team and an outline programme was designed. This comprised of two three hour sessions held at an easily accessible venue familiar to members of the PPI group. The course included theories behind qualitative research methodology and methods, what is coding and how to code independently and as part of a research team using Thematic Analysis. A mock research question was generated and two mock interviews were completed, audio recorded and transcribed verbatim. This provided participants with real life experience of coding data. The session was positively reviewed and said to be interesting, enjoyable and provided a good overview of qualitative analysis. One of the PPI partners subsequently went onto code data with a researcher working on a funded research study, and has reflected on both the training sessions and the subsequent analysis of the data. These reflections have been supplemented by reflections of the researcher who worked alongside the PPI, revealing that the process challenged perspectives and helped them view data through a service users eyes. A positive working relationship was central to this.

**Conclusions:**

Feedback suggests that the training enabled PPI partners to become active members of the research team in qualitative data analysis. There is a need for further research into the optimal amount of training needed by PPI’s to participate as partners in qualitative analysis.

## Background

NHS guidance states that service user involvement should exist at every stage of the research process [1] and there are increasing examples of this involvement throughout the research cycle, particularly in planning, designing and in data collection. However it has been argued there are less examples of Patient and Public Involvement (PPI) in analysing and interpreting qualitative data [2, 3]. Involving PPI partners in qualitative analysis has been recognised as particularly valuable as they can draw on their own experiences to make sense of the data [4], and their involvement has been recognised as a means of improving the quality, robustness and validity of the analysis [5, 6]. Although PPI in health and social care research has been on the increase, it has been noted that research in partnership with older people has been slower to develop than with other service user groups and have often been bypassed by these initiatives [7]. Clough et al. [8] reported that one of their greatest frustrations was finding older PPI partners who had been involved in other research studies. Three studies have been identified where older PPI partners were involved in qualitative analysis [4, 7–10].

An essential requisite of PPI in any aspect of the research process is the provision of appropriate training to equip them with the necessary knowledge and skills, without which, the ‘research product’ will be compromised [11, 12]. Indeed one of the eight principles of successful PPI in NHS research has been identified as training [13]. Although training has been recommended for PPI in research little is known about what training is needed [14]. A recent mapping exercise was conducted to identify such training initiatives in health and social care, and this revealed just 26 initiatives across England, with 12 of these initiatives providing training on analysing and interpretation of data. The training across these initiatives was diverse in respect of style, content and length of provision, with training provided on a single day through to training spread out over several months [11]. One course has gone further to provide training to service users over two academic terms on qualitative interviewing and analysis, culminating in a validated university certificate in research methods [8]. It is less clear therefore on the quantity and content of training that would be beneficial for PPI partners participating in qualitative analysis.

Another concern raised in the literature is how to strike a balance between increasing PPI research knowledge and skills, enabling their participation as partners, whilst not professionalising their involvement [7, 9, 14]. There is a need therefore to explore appropriate training education for PPI partners partaking in qualitative analysis that prepares them to work alongside research partners, whilst at the same time, retaining their unique contribution as service users.

Dewar [15] has called for more opportunities for the sharing of experiences about the process of involving older people in research. This paper therefore describes one example of qualitative data analysis training provided to a group of older PPI partners. It incorporates a reflective account, from the researcher and service user perspective, of implementing this training into research practice. Implementation of research skills into practice has been identified as a key outcome of service user training [11].

In 2017 the primary author, Alison Cowley, was awarded a Clinical Doctoral Research Fellowship funded by Health Education England and the National Institute for Health Research to explore how healthcare professionals define and apply the concept of ‘rehabilitation potential’ in frail older people in the hospital setting and to develop and test an assessment tool for use in clinical practice. The fellowship included funding to train members of the public to code focus group data alongside members of the research team to provide a differing perspective on analysing research data.

The aim of this article is to describe the development and implementation of the qualitative data analysis training programme and the experiences of the academic researchers and ‘lay’ researchers in data collection and analysis.

### Qualitative data analysis training

A small training team, comprised of staff from the Division of Rehabilitation, Ageing and Well-being (JD, AC, MC) reviewed existing literature on PPI in qualitative data analysis and an outline programme was developed to meet the needs of the identified participants. The training team consisted of two experienced qualitative researchers (JD and AC), a clinical nurse (MC), and a Professor experienced in the development of complex rehabilitation interventions (PL). In view of the limited availability of qualitative analysis training for older PPIs, advice on the content and format of training was sought from TW, who had previously published work around older lay researchers conducting qualitative data analysis following training [10]. Ideally a member of the local PPI group would have been involved in the design and content of the course, but as PPI experience of qualitative analysis was limited, and was the motivating factor behind developing the course, this was not feasible The course was designed to specifically train members of the Dementia, Frail Older People and Palliative Care PPI Advisory Group at the University of Nottingham, who comprise of older adults. The group, established in 2012, consists of 20 members of the general public who advise on research priorities, study design, ethical considerations and dissemination of studies at the University of Nottingham and the wider health community. Many of the group are co-applicants for large nationally funded studies. Prior to the training programme, members had no experience of qualitative data analysis but were keen to develop these skills. A formal evaluation of pre and post course knowledge and skills was not completed. They attended the group on a voluntary basis.

The course was held over two sessions of 3 h duration in February and March 2018 at an easily accessible venue in the University of Nottingham familiar to members of the PPI group. This was to ensure that they felt at ease during the session, and is in line with other PPI partners training programmes around research [11]. Seven members of the group attended the first training session with five participants attending both sessions. Again attrition is common amongst other PPI partners training programmes [11].

The first training session included taught sessions of the theory behind the use of qualitative research methodology and methods, what is coding and how to code as an independent researcher and as part of the wider multi-disciplinary team. This provided participants with a basic introduction into the epistemology associated with the qualitative paradigm, strengths and weaknesses enabling the appreciation as to why researchers use qualitative methods. Participants were taught the principles behind coding qualitative data and more specifically how to code data using Thematic Analysis. This analytic method was selected as it has been recognised as a foundational method for learning qualitative analysis skills [16], and a method that has been used with a variety of service user groups [3, 4, 17]. A clear step-by-step guide was sought to provide structure to the Thematic Analysis process. Braun and Clarke’s [16] Thematic Analysis Guide met this requirement. This six phase guide was used to structure the training sessions.

The training team developed a ‘mock’ research question titled “What factors affect medication adherence in adults living in the community setting?” This topic was chosen as it was felt that potential participants would have experience of either taking medications themselves or supporting others in medication adherence but was unlikely to be too controversial, providing a ‘safe’ training topic and exercise. Two ‘mock’ semi-structured interviews were completed and audio recorded for this training by the team. Participants on the course were asked to listen to the audio recording of the first interview to familiarise themselves with the data and were then given the transcript (transcribed by the team) to read through and make reflective notes. This was then discussed with the group followed by time to read and discuss a pre-prepared coded transcript of the interview. The second interview audio recording was played to the group and participants were given the second transcript to independently code at home in preparation for the second session. Prior to the close of the session, participants were advised that they could contact the team if they required further support whilst coding the transcript at home.

The second training session, held 2 weeks later, provided the participants with the opportunity to share their experiences from the coding exercise and to discuss the codes generated from the two interview transcripts. The training then progressed to look at how codes are sorted into potential themes. The participants were separated into two groups and provided with cards, each card labelled with the code name and its descriptor. The participants then sorted the cards into potential theme piles, and then discussed their constructed themes in relation to the research question. The session concluded with reflections on the course and an opportunity to ask the trainers questions.

### Reflection on qualitative data analysis training

Five participants on the course provided feedback on the course evaluation form and through email correspondence. Feedback has been anonymised and included in this commentary paper with permission from the participants. Overall, the course was said to be interesting and enjoyable, providing participants with an introduction into qualitative data analysis. One participant stated that:

*“Previously I knew nothing of analysing qualitative data and this ignorance had become an increasing concern for me as I have been and am still the lay co-applicant on several studies and attend management meetings where discussion included such topics…. The course was an excellent introduction to the topic. With two sessions and homework it gave me an opportunity to flesh out my understanding with practical exercises.” [Participant One].*

The use of ‘mock’ interviews to provide participants with real life examples were positively received, highlighting the challenges that all researchers face when coding data autonomously and as part of a team:

*“I see you need to do it [code] several times…and it was surprising that we all produced surprisingly similar results from us all.”* [Participant Two].

Despite the condensed nature of the training course, compared to many other research training initiatives which can span from anything from a single day to 28 days [11], participants described an increased confidence when dealing with qualitative research data such as focus groups and interviews:

*“I did a mature degree using qualitative research, and whilst was well tutored and the degree was a good one, the level of QD [qualitative] analysis was nowhere near as intricate or sophisticated as your course.”* [Participant Three].

Whilst the course was primarily aimed to train PPI partners in coding qualitative research data, it was reported to also increase their confidence in questioning choices made by researchers:

*“The questions I ask will be different, more incisive and detailed… Did they build up themes, codes and a suitable QD strategy? Researchers coming in front of PPI panels often tend to concentrate on general procedures, using lay language, ethics, accuracy of paperwork etc., when really the PPI panel should be more concentrated on the background and quality of QD implementation, as this really is the central issue of the research.”* [Participant Three]

One of the key benefits of training PPI partners has been identified as increased confidence, motivation and skills to be actively involved in research activities [11]. The condensed nature of the training course did not appear to diminish this positive outcome.

The participants on the course began to reflect on the additional contributions that PPI partners could bring to the research process, moving from a tokenistic involvement towards true co-production. Training was viewed as a:

*“Genuine commitment to enhance our skills as PPI volunteers, replenishing our enthusiasm for being part of the team” [Participant One].*

### Implementing training into practice

As part of a PhD study, five focus groups were co-facilitated by AC and MK (PPI member) consisting of 28 participants. The discussions were audio recorded and the data was transcribed verbatim. Ethical approval for the focus groups was obtained. The Framework Approach, which sits within the broad family of thematic analysis, was used to analyse the data [18, 19]. It is recommended for collaborative multi-disciplinary research [19] involving seven distinct stages designed to provide a robust and transparent method of qualitative data analysis. Table 1 outlines the seven steps of the Framework Approach and PPI in each stage. Both AC and MK kept reflective diaries during data collection and analysis and excerpts from these documents will be shared to highlight the challenges and opportunities for collaborative data analysis.

**Table 1:** Framework Analysis Approach with PPI

Stage	Description	PPI
1. Transcription of data	Audio data is converted to a written transcript	None
2. Familiarisation	The audio recording is subjected to repeated listening and the transcript repeated reading	MK repeated reading of all five transcripts
3. Coding	The transcript is read line by line and a code or label is applied by the researcher	MK developed codes for 2 transcripts
4. Development of analytical framework	Where research team meet to discuss and compare their codes and agree on a framework of codes to be applied to subsequent transcripts	MK met with AC and discussed codes and together developed framework
5. Application of framework	The working analytical framework is applied to remaining transcripts. New codes/labels are added to the framework which is further refined	MK applied framework to the remaining 3 transcripts and reviewed 2 initial transcripts against the framework
6. Charting	A summary of the data is produced from which codes are built into themes	MK reviewed data summary
7. Interpreting	Expand and interpret themes, and develop an analytical understanding	MK and AC discussed interpretations and overarching themes.

### PPI reflections

Having volunteered to support Alison with her research project which would involve using the coding training we took part in, I was curious to see how it worked in practice. When you attend a focus group, either as a member or facilitator, you come away with an opinion on what was said and what main points were raised. Having coded the date from these focus groups you realise what would be missed if you didn’t look closely at the written transcripts. It takes time to code data; one read through is not enough, but the main points raised gradually begin to gel as you become more familiar with your codes and what you are reading. I found that I had to be especially aware of any bias slipping in and the positives and negatives of bringing my own experiences to the table. I was surprised how much more relevant detail was uncovered using this technique.

Having coded two transcripts, I met with Alison and was surprised/relieved/amazed to discover that the points I had identified were basically the same as hers. That and the training gave me the confidence to complete the remaining focus groups and that my views were as equally valid in the analysis. As a PPI partner I felt my interpretation should come from that perspective. This is an essential part of the research process and with the right training and support, PPI representatives can positively contribute to analysis of research data. Professional researchers automatically take into account the restraints of their working environment, whereas ‘lay people’ are generally unware of these or choose to ignore them and we can therefore look at findings with a more open mind.

### Researcher reflections

Involving PPI partners in co-production of qualitative data analysis has led to me challenge my own perspectives and beliefs. Researchers or clinicians view the world, their practice and data through a ‘professional’ lens. We frequently recruit patients, carers, families and service users to studies but their voice can then be lost when data is analysed. We are embedded in the process, our research question and despite being reflective, we still see the world as clinicians or researchers. Co-facilitating and analysing the data with MK led me to challenge my assumptions not only around the clinical practice of rehabilitation assessments but also the notion of good outcomes. During data coding, I found my focus was drawn towards very medical/technical terms and how rehabilitation assessments were operationalised whereas MK was good to drawing attention towards the patient and carer experience. By discussing these two perspectives we were able to develop a number of cross cutting themes such as communication, clinical staff training and expertise and supporting family member’s involvement in rehabilitation which would have not featured strongly in the final analysis without lay involvement. Other studies have similarly found that lay involvement has identified themes which would otherwise have been missed without their input in the analysis process [6, 17].

In order to achieve these insights it was essential to develop an open and honest relationship where MK was supported and encouraged to challenge and question. We conversed frequently via email but this was supplemented with regular face-to-face meetings in a comfortable environment; MK’s house, so we could talk through our findings and develop a joint understanding of the data. I found that I had to clearly articulate the codes and themes and adopt a non-jargonistic terminology and language. This reflected concerns that although they made sense to me, as a researcher and clinician, they may not have resonated with the understanding of members of the public. By providing a clear and unambiguous coding framework and articulating the process of data analysis, greater rigour and transparency was introduced into the study.

When coding data a plethora of interesting findings may emerge and I found that both I and MK were guilty of going off topic at times. The study sought to understand what was meant by the clinical term ‘rehabilitation potential in older people living with frailty’, why this was used and how it was assessed. The resulting focus groups led to lively discussions around outcomes of rehabilitation and whilst interesting, they did not directly address the research question. Through an open dialogue and regular meetings we were able to mutually steer our data analysis back towards the research question, parking our extraneous musings for a later date.

## Conclusion

In order to ensure that research interventions are designed which embrace and understands everyone’s needs, it is essential that members of the public are involved in all stages of the research process, not just the study design and dissemination. This reflective account revealed that designing and providing a qualitative data analysis training programme for members of a University PPI Advisory Group was feasible and well received by participants. Feedback revealed that this training increased participant knowledge and confidence. This is in keeping with studies conducted with PPI partners [7, 10, 11]. The training has since enabled two members of the group to take part in qualitative data analysis as part of the research team, providing differing perspectives, viewpoints and a voice for those whom the ultimate intervention is designed for. These strengths have been recognised by studies which have evaluated the impact of PPI partners upon research studies [7, 10, 11]. Since the completion of the course, MK (PPI partner) has been involved in a qualitative analysis course for post-graduate students and researchers, where she shared her experiences of conducting data analysis and working as a member of a research team, providing advice and guidance for others aspiring for greater PPI. Her perspective and experiences were positively evaluated by course attendees. Research has shown that training and subsequent involvement in qualitative research can provide a platform for participants to move onto complete other such projects [10, 11].

In order to achieve an effective working relationship, ongoing support and guidance is essential, where all parties respect and value each other’s knowledge, opinions and experiences whilst working towards answering a specific research question. Involving PPI partners in analysing qualitative research data provides a differing perspective, challenging researchers’ assumptions and positions. It has the potential to enhance the analysis process. Another member of the University PPI Advisory Group has also gone onto conduct qualitative analysis as part of a Process Evaluation embedded into a large Randomised Controlled Trial.

It should be recognised that this commentary provides a reflective account of a small scale training programme and therefore the generalisability of the findings are limited.

### Implications for the future

In light of the above limitation, further research is needed to understand the optimal amount and type of training provided for PPI partners to support their active involvement in qualitative data analysis. Future training courses for qualitative data analysis by older adults should be co-designed by those they are intended to support, thus ensuring that course content and delivery are appropriate and fit for purpose. Further research is also recommended to explore the type and amount of training professional researchers require to effectively integrate PPI partners into the research process and fully realise the potential of their contributions.

## Data Availability

Data sharing is not applicable to this article as no dataset were generated or analysed during the current study.

## Abbreviations

HEE: Health Education England
NIHR: National Institute for Health Research
PPI: Patient and Public Involvement
